# Genomic Signatures of Independently Evolved Walking-Associated Locomotion in Fishes

**DOI:** 10.3390/biology15211990

**Published:** 2026-11-03

**Authors:** Qin Wang, Bao Wang, Chenglong Zhu, Huishan Yue, Deqian Fan, Wenjie Xu, Jiangmin Zheng, Jianzhong Ling, Liulan Zhao, Song Yang, Xiaojing Song, Ling Tan

**Affiliations:** 1 Shaanxi Key Laboratory of Qinling Ecological Intelligent Monitoring and Protection, School of Life Science and Technology, Northwestern Polytechnical University, Xi’an 710072, China; lavender_412@163.com (Q.W.); wangbao0716@163.com (B.W.); zhuchenglong96@gmail.com (C.Z.); monyhs@mail.nwpu.edu.cn (H.Y.); daisybluekano@gmail.com (D.F.); wenjiexu1996@gmail.com (W.X.); zhengjiangmin@mail.nwpu.edu.cn (J.Z.); 2 Department of Medical Oncology, Cancer Center, Key Laboratory of Tumor Molecular Diagnosis and Individualized Medicine of Zhejiang Province, Zhejiang Provincial People’s Hospital, Affiliated People’s Hospital, Hangzhou Medical College, Hangzhou 310014, China; 3 Department of Basic Medicine, School of Medicine, Jianghan University, Wuhan 430056, China; 4 East China Sea Fisheries Research Institute, Chinese Academy of Fishery Sciences, Shanghai 200090, China; lingjz@ecsf.ac.cn; 5 Fisheries College, Sichuan Agricultural University, Chengdu 611130, China; zhaoliulan2007@163.com (L.Z.); ysys210@hotmail.com (S.Y.)

**Keywords:** spiny red gurnard, pectoral fin ray, comparative genomics, convergent evolution, locomotor adaptation

## Abstract

Walking-associated locomotion has evolved independently in several fish lineages through distinct anatomical and biomechanical mechanisms. The spiny red gurnard (*Chelidonichthys spinosus*) provides an experimentally tractable model for dissecting the genetic basis of walking-associated locomotor specialization. We assembled a high-quality chromosome-level genome of the spiny red gurnard and performed a comparative genomic analysis across five walking-associated fish lineages. An integrated protein–protein interaction (PPI) network revealed high clustering coefficients and significant enrichment in fin development and locomotion pathways, indicating candidate functional similarity of lineage-specific genes at the protein interaction level. Shared positively selected genes (PSGs), including *jph2*, *ccdc80*, *lrrc7*, and *ap3b2*, were identified as candidate genes potentially associated with neuromuscular function. Specifically, in the spiny red gurnard, *cdh23*, showed lineage-specific positive-selection signals and amino acid changes, suggesting a potential contribution to mechanosensory adaptation. Furthermore, we identified changed conserved noncoding elements (CNEs) in each lineage, with some showing convergent changes near *wnt3* and *cyp26a1* in specific species pairs. A species-specific CNE deletion in the *tax1bp1b* intron was tested in HEK293T cells using sequences from zebrafish and spiny red gurnard; the two sequences showed different heterologous reporter activities. Overall, our analyses provide exploratory evidence for a dual pattern of genomic variation potentially underlying walking locomotor evolution: functional association at the protein network level coupled with predominantly lineage-specific yet occasionally convergent regulatory changes. This work also offers insights into environmental adaptation in fishes and a valuable genomic resource for studying convergent evolution.

## Simple Summary

Several fish lineages have independently evolved walking-associated locomotor systems through different anatomical and biomechanical mechanisms, providing a useful framework for studying convergent evolution. However, the genetic mechanisms underlying this repeated locomotor adaptation remain unclear. Here, we generated an improved genome assembly of the spiny red gurnard and performed comparative genomic analyses with four other independently evolved walking-associated lineages (five species, including two frogfish species) and fourteen non-walking species, totaling 20 species. We constructed a protein–protein interaction network and found that proteins encoded by positively selected genes from different lineages were associated with multiple processes related to fin development and locomotion. We identified changed conserved non-coding elements across different lineages of walking fishes, with their numbers varying substantially from 1245 to 21,663. In the spiny red gurnard, we further identified a positively selected gene potentially associated with mechanosensation and a species-specific regulatory alteration that showed reduced reporter activity in a cell-based assay. Our findings highlight candidate-shared and lineage-specific genomic signatures associated with walking locomotor evolution, including functional associations at the protein network level and predominantly lineage-specific regulatory changes.

## 1. Introduction

Exaptation, the co-option of preexisting traits for new functions, is a fundamental concept in understanding morphological innovation and has occurred in many organisms, sometimes serving as an outcome of organ specialization for adaptation [1]. In the evolutionary history of vertebrates, the emergence of paired appendages has greatly enhanced locomotor and adaptive capabilities, and further specialization of the pectoral fins in certain fishes provides an ideal model for studying exaptation [2,3,4]. Among them, the family Triglidae, commonly known as gurnards or sea robins, provides a striking example of this process. These marine organisms have markedly transformed their pectoral fins not only for swimming but also into sophisticated sensory and locomotor appendages, enabling them to “walk” on the seabed and detect chemical cues in their environment [5,6]. Our previous study revealed the molecular basis of chemosensation in the pectoral fins of the spiny red gurnard (*Chelidonichthys spinosus*), mediated by an ancient olfactory receptor homolog *srgor* [7]. Recent studies have begun to resolve the developmental basis of these specialized appendages. In particular, Herbert et al. [8] identified *tbx3a* as a major determinant of sea robin (*Prionotus carolinus*) leg development and demonstrated its roles in leg morphology, associated neural structures, sensory papillae, and digging behavior. Together with evidence for the mechanosensory and chemosensory specialization of sea robin legs [5], these findings have substantially advanced our understanding of the developmental and sensory biology of triglid pectoral appendages. However, whether these developmental mechanisms are shared across independently evolved walking-associated fish lineages remains unclear.

Previous studies have shown that these specialized fins possess complex mechanical properties similar to those of the limbs of vertebrates, providing robust support and dynamic movement [9]. Walking-like locomotion in fishes is anatomically diverse and can involve different appendicular structures. For example, pelvic fins contribute substantially to substrate-based locomotion in skates and lungfishes [10,11], whereas the shuttles hoppfish (*Periophthalmus modestus*) uses modified pectoral fins for substrate-supported terrestrial locomotion [12], and the striated frogfish (*Antennarius striatus*) uses elbow-like pectoral fins to move along the benthic substrate [13]. In contrast, terrestrial locomotion in the climbing perch (*Anabas testudineus*) relies primarily on opercular support and axial body movements, with pectoral fins contributing mainly to support and stabilization rather than propulsion [14]. The North African catfish (*Clarias gariepinus*) employs a combined axial–appendicular mechanism in which body bending is coordinated with the robust pectoral-fin apparatus [15]. Together with the free pectoral rays used for benthic locomotion in the spiny red gurnard, these species therefore represent independently evolved walking-associated locomotor systems rather than anatomically equivalent manifestations of a single pectoral-fin phenotype (Table S1). This phenotypic diversity provides an opportunity to test whether distinct locomotor architectures are nevertheless associated with shared or lineage-specific genomic signatures.

Classical evolutionary biology indicates that similar phenotypes can arise either from shared genetic mechanisms or through distinct molecular routes [16,17]. In evolutionary developmental biology, morphological evolution is often achieved through changes in cis-regulatory elements rather than alterations in protein-coding sequences [18,19]. Recent comparative genomic work has further demonstrated that recurrent changes in conserved noncoding elements can accompany convergent appendage evolution across distantly related fish lineages. For example, Chen et al. [20] identified repeated deletions of conserved regulatory sequences associated with recurrent pelvic- and caudal-fin reduction across diverse percomorph fishes. Despite these advances, it remains unclear whether independently evolved walking-associated appendages across divergent teleost lineages show shared genomic signatures or instead arise predominantly through lineage-specific molecular changes. In particular, the relative contributions of protein-coding evolution, regulatory sequence variation, and candidate convergence at the level of protein interaction networks have not been systematically compared across multiple walking-associated fish lineages. Addressing this question requires an explicitly cross-lineage comparative framework that distinguishes shared signals from lineage-specific changes.

In this study, we generated a new de novo genome assembly of the spiny red gurnard using the same raw reads as Li et al. [7]. The new assembly contains only seven gaps, compared with 25 gaps in the previous draft assembly [7], suggesting an assembly with higher contiguity for downstream analysis. Leveraging this enhanced assembly together with publicly available reference genomes, we performed a comprehensive comparative genomic analysis of five walking-associated fish lineages exhibiting independently evolved walking-associated locomotor behaviors: the spiny red gurnard, shuttles hoppfish, striated frogfish, climbing perch, and North African catfish. This comparative framework enabled us to investigate whether these distinct walking-associated locomotor systems exhibit shared or lineage-specific genomic signatures. We first identified PSGs in each lineage and constructed an integrated PPI network to assess similarities in functional organization among candidate PSGs. We then examined the evolutionary dynamics of CNEs adjacent to fin-development genes to identify shared and lineage-specific regulatory changes. Using this framework, we investigated whether independently evolved walking-associated fish lineages exhibit shared or lineage-specific signatures at the coding, protein-network, and regulatory levels. Our analyses identify candidate patterns of functional similarity in protein association networks together with predominantly lineage-specific, and in some cases shared, changes in conserved noncoding elements. These results provide candidate genomic signatures associated with walking-related appendage evolution rather than evidence for a single conserved molecular mechanism.

## 2. Methods

### 2.1. Genome Assembly, Quality Assessment and Annotation

Procedures for DNA extraction, library preparation, and sequencing followed standard protocols and are detailed in a previous study on the same species [7]. No new sequencing was performed in this study; all specimens and raw sequencing data were obtained from Li et al. [7], where specimen collection, ethical approval (approval no. 202101025), and sequencing procedures are described in detail. Raw short sequencing reads were filtered using fastp v.0.20.0 [21]. The genome size was estimated via a k-mer-based method (k = 27) using the kmerfreq module within SOAPec v.2.0 [22]. The genome was assembled *de novo* from PacBio HiFi reads, with Hi-C reads integrated to anchor contigs into chromosome-level scaffolds using Hifiasm v.0.25.0 (r726) [23] with parameters “--dual-scaf --telo-m CCCTAA”. The genome assembly was then validated, grouped, sorted and anchored with the Hi-C reads to generate pseudo-chromosomes, by using Juicer (version 1.6) [24] and 3D-DNA (version 180114) [25]. In addition, we utilized Juicebox (v.1.11.08) (https://github.com/aidenlab/Juicebox, accessed on 25 October 2026) to visualize and check the Hi-C data. We filled gaps in the pseudo-chromosomes using HiFi reads (aided by TGS-GapCloser v1.2.1) [26]. The completeness of the final genome assembly was assessed using BUSCO v.5.4.3 [27] with the actinopterygii_odb10 database. In addition, the reference-free k-mer-based tool Merqury v.1.3 [28] was used to compute a quality value (QV) score for the assembly.

Repetitive sequences were annotated using RepeatModeler v.1.0.11 [29] and RepeatMasker v.4.0.7 [30] against RepBase. Protein-coding genes were annotated by integrating *de novo* (Augustus v.2.5.5 [31] and ANNEVO v.2.1 [32]), transcript-based (SPAdes v.3.1.1 [33] and TransDecoder v.5.5.0 [34]), and homology-based (miniprot v0.12-r237 [35]) approaches and consolidated using EVidenceModeler v.1.1.1 [36] to generate the final consensus gene annotation.

### 2.2. Single-Cell RNA-seq Analysis

Single-cell RNA-seq data were obtained from Li et al. [7] (accession: SRR30484486, SRR30484490, SRR30484501 and SRR30484502) and processed exactly as described in that study. In brief, quality control, normalization, clustering, dimensionality reduction, and cell-type annotation were performed using the same parameters and pipelines as in Li et al. [7]. Candidate gene expression across cell types was visualized and statistically compared following the same methods. Detailed parameters are provided in Li et al. [7].

### 2.3. Comparative Genomics Analysis

#### 2.3.1. Phylogenetic Analysis and Divergence Time Estimation

To reconstruct the phylogenetic relationships among the five walking-associated fish lineages, we performed a phylogenomic analysis using 20 ray-finned fish species (Table S2). For each gene with multiple annotated transcripts, we selected the longest transcript as the representative isoform and retained its corresponding protein and CDS sequences. One-to-one orthologous genes were identified using DIAMOND v.2.1.4 [37] and the reciprocal best-hit (RBH) criterion, with zebrafish as the reference. Reciprocal matches were integrated across the sampled species, with one representative gene sequence per species retained in each group. This yielded 3743 RBH-defined one-to-one orthologous groups. The protein sequences in each orthologous group were aligned using MAFFT v.7.471 [38]. Maximum likelihood gene trees were constructed using IQ-TREE v.1.6.12 [39] with the parameters: -m MFP -alrt 1000.

The gene trees were integrated using ASTRAL v.5.7.1 [40] to infer a coalescent species tree. PAL2NAL v.14 was used to map the MAFFT protein alignments to the corresponding CDS sequences, producing codon alignments that preserved the reading frame. Unreliable alignment regions were removed using Gblocks v.0.91b in codon mode (-t=c). Sequences containing frameshifts, premature stop codons, or ambiguous codons were excluded before codon alignment. Divergence times were estimated from these filtered codon alignments using the MCMCTree module within the PAML v.4.9j package [41] with three calibration points from the TimeTree database [42]. The syntenic relationships between the spiny red gurnard and humphead wrasse genomes were examined using the JCVI v.1.3.3 [43].

#### 2.3.2. Positive Selection

Selection analyses used the same filtered codon alignments of the RBH-defined one-to-one orthologs described in Section 2.3.1. These alignments and the species tree were analyzed using codeml in PAML v.4.9j [41], with seqtype = 1, CodonFreq = 2, and icode = 0. Each of the five walking-associated fish lineages, spiny red gurnard, shuttles hoppfish, striated frogfish, climbing perch, and North African catfish, was designated as the foreground branch in a separate analysis. For the frogfish lineage, the terminal branches of both striated frogfish and warty frogfish were labeled as foreground in the same analysis.

The free-ratio model (model = 1, NSsites = 0) estimated branch-specific ω = dN/dS values. The branch model compared a one-ratio model (model = 0, NSsites = 0) with a two-ratio model (model = 2, NSsites = 0) that assigned separate values to foreground and background branches. These analyses assessed lineage-specific differences in average selective pressure. PSGs were identified using the branch-site test (model = 2, NSsites = 2). For the foreground positive-selection site class, we set ω = 1 in the null model (fix_omega = 1, omega = 1) and estimated ω in the alternative model (fix_omega = 0).

Branch-model comparisons used a chi-square reference distribution with one degree of freedom. The branch-site test used the same distribution as a conservative reference. Gene-level branch-site *p* values were adjusted using the Benjamini–Hochberg procedure across all valid gene tests, separately within each foreground lineage. Genes with BH-adjusted *p* < 0.05 were classified as FDR-supported PSGs. Genes with raw *p* < 0.05 but BH-adjusted *p* ≥ 0.05 were labeled as exploratory candidates and distinguished from FDR-supported PSGs. Table S3 reports model log likelihoods, LRT statistics, raw and adjusted *p* values, and estimated ω values. Positively selected amino acid sites were inferred using the Bayes Empirical Bayes (BEB) procedure implemented in codeml, and sites with posterior probability > 0.9 were considered candidate positively selected sites. Codeml analyses with unreliable or non-convergent parameter estimates were excluded from the FDR-supported PSG set.

AlphaFold3 [44] was used to predict the three-dimensional structures of proteins encoded by candidate PSGs. Candidate positively selected amino acid sites were mapped onto the corresponding predicted structures. Their potential structural and functional implications were assessed from their domain locations and the nature of the amino acid substitutions.

#### 2.3.3. Analysis of Conserved Noncoding Elements (CNEs)

Taxon sampling for the CNE analysis differed from that used for phylogenomics and positive selection. The latter used 20 ray-finned fish species for orthology inference, species-tree reconstruction, and lineage-specific selection analyses, whereas the CNE analysis used a separate 21-species teleost dataset (Zebrafish, Channel catfish, North African catfish, Shuttles hoppfish, Zig-zag eel, Climbing perch, Humphead wrasse, Spiny red gurnard, Three-spined stickleback, Tiger puffer, Warty frogfish, Striated frogfish, Japanese eel (*Anguilla japonica*), Reeves moray (*Gymnothorax reevesii*), Electric eel (*Electrophorus electricus*), Armored catfish (*Cranoglanis bouderius*), Corkwing wrasse (*Symphodus melops*), Korean rockfish (*Sebastes schlegelii*), Lumpfish (*Cyclopterus lumpus*), Orange-throat darter (*Etheostoma spectabile*) and European perch (*Perca fluviatilis*)) with broader and denser sampling within Teleostei, including additional early-diverging and acanthomorph lineages. This design improved whole-genome sequence comparability and conservation inference and helped distinguish lineage-specific CNE changes from differences caused by deep phylogenetic divergence (Table S2).

Whole-genome comparisons were performed across the 21-species CNE dataset using the zebrafish GRCz11 assembly as the reference. Pairwise genome alignments were generated using LAST v.1282 [45] with lastdb -uMAM8 and lastal -pHOXD70. The pairwise alignments were progressively combined using Multiz v.11.2 [46] according to the phylogenetic topology corresponding to the CNE taxon set, shown below. The dynamic-programming radius was set to R = 30 and the minimum output width to M = 1. At each merging step, the alignment containing the zebrafish reference row was supplied as the first input, with v = 1 to keep that row fixed. MAF headers were retained, and repetitive sequences were soft-masked before alignment. 

Fourfold-degenerate sites were extracted from the multispecies CDS alignments using the zebrafish coding annotation, excluding sites associated with incomplete codons, ambiguous bases, or alignment gaps. A neutral substitution model was estimated using phyloFit in PHAST v.1.5 [47] with the CNE species-tree topology fixed. The REV nucleotide substitution model was used, with --subst-mod REV --tree <species tree>. Branch lengths, equilibrium nucleotide frequencies, and substitution-rate parameters were estimated by maximum likelihood to generate the neutral model for conservation analysis.

Conserved regions were identified using phastCons v.1.5 [48] and the estimated neutral model, with --target-coverage 0.3, --expected-length 45, and --rho 0.3. These parameters specified the prior conserved-state coverage, expected conserved-element length, and branch-length scaling of the conserved model relative to the neutral model, respectively. Conserved regions were obtained using --most-conserved. Regions overlapping annotated CDS in the zebrafish GRCz11 assembly were excluded, and candidate CNEs with reference lengths greater than 30 bp were retained.

SlideVar pipeline [49] was used to screen for sequence changes within the candidate CNE alignments. It was run with 20 bp windows, requiring at least 18 reference matches in background windows and no more than 12 matches in the focal lineage, while allowing up to two background species to fall below the background-conservation threshold, corresponding to -w 20 -con 18 -div 12 -bn 2. For each walking-associated fish lineage, CNEs meeting the divergence thresholds were compiled into a species-level candidate set. These sets were then integrated to identify changes shared by at least two walking species and changes detected in only one focal species.

Based on the zebrafish GRCz11 annotation, genes with transcription start sites within 1 Mb upstream or downstream of candidate CNEs were considered candidate associated genes for downstream screening. This proximity-based assignment does not establish enhancer–target linkage. Candidate deletions were distinguished from assembly gaps and alignment failures by inspecting read support and assembly continuity. Only deletions with read-level support or consistent assembly evidence were retained. Subsequent analyses focused on genes involved in growth and development.

#### 2.3.4. Construction of the Protein Interaction Network

A protein–protein interaction network was constructed using PSGs from five walking-associated fish lineages. The PPI network was constructed using the nominal candidate PSGs (raw *p* < 0.05) identified across the five lineages. Protein sequences were mapped to the STRING v.12.0 [50] using zebrafish (Danio rerio) as the reference organism. Identifier mapping was performed using the dbOrtho mapping model implemented in STRING. The minimum interaction confidence score was set to 0.4 (medium confidence), and the maximum observed score was 0.998. All available evidence channels were considered, including experiments, databases, co-expression, neighborhood, gene fusion, co-occurrence, and text mining. No additional interactors were permitted beyond the input PSG-encoded proteins. The resulting network was used for visualization and subsequent enrichment analyses. GO enrichment analysis was performed using biological process (BP) terms, and KEGG pathway analysis was performed separately, both using the g:Profiler tool (https://biit.cs.ut.ee/gprofiler/gost, accessed on 25 October 2026) [51]. The tested background consisted of all genes in the STRING-mapped network component; adjusted *p* values were calculated using g: Profiler with Benjamini–Hochberg correction.

### 2.4. Dual-Luciferase Reporter Assay

HEK293T cells (YC-A007, Ubigene, Guangzhou, China) were selected for in vitro regulatory activity experiments. HEK293T cells were cultured in Dulbecco’s modified Eagle’s medium (DMEM) (Catalog No. C11995500BT; Gibco, Thermo Fisher Scientific, Santa Clara, CA, USA) supplemented with 10% fetal bovine serum (Catalog No.10091148; Gibco, CA, USA). First, the sequences of the identified candidate CNEs were cloned into and inserted into the pGL4.23-promoter vector (Catalog No. E8411; Promega, Madison, WI, USA), which was digested by KpnI (Catatlog No. R3142V, NEB, Ipswich, MA, USA) and NheI (Catalog No. R3131V, NEB, MA, USA) in the upstream region of the luciferase gene. All reporter plasmid constructs were subsequently transfected using PEI (Catalog No. 40815ES03; Yeasen, Shanghai, China), and the Renilla luciferase vector pGL4.74 (Catalog No. E6921; Promega, Madison, USA) was used as an internal control. Specifically, 400 ng of reporter plasmid and 20 ng of pGL4.74 were co-transfected using PEI at a DNA:PEI ratio of 1:1. Luciferase expression was subsequently monitored with a dual luciferase assay (Catalog No. E1910; Promega, Madison, WI, USA) 48 h after transfection. The assay was performed in six independent experiments, each with eight technical replicates per treatment. Data are presented as mean ± SD. Statistical significance was evaluated using Student’s *t*-test. The results are expressed as luciferase/Renilla ratios with vectors carrying putative enhancers relative to the ratio with the empty pGL4.23 vector, and statistical significance analysis was performed using the normalized data. The complete insert sequences, lengths, cloning primers, verification primers, and sequencing verification information are provided in Table S4.

## 3. Results and Discussion

### 3.1. Chromosome-Level Genome Assembly and Phylogenomic Analysis Across Walking Fish Lineages

PacBio HiFi, Hi-C, and paired-end short-read sequencing were performed for spiny red gurnard, generating approximately 30 Gb of PacBio HiFi reads, 100 Gb of Hi-C reads, and 100 Gb of Illumina paired-end short reads. We performed a new de novo assembly using the same raw reads as Li et al. [7]. After stringent filtering to remove low-quality and contaminant sequences, we assembled a high-quality genome with a total size of 669.24 Mb, consisting of 158 scaffolds (contig N50 = 26.66 Mb; scaffold N50 = 28.0 Mb). Using Hi-C data, 96.1% of the assembly was anchored onto 24 pseudo-chromosomes (Figure S1). The assembly completeness evaluated by BUSCO (actinopterygii_odb10) reached 98.6%. Merqury analysis [28] generated a QV score of 59.50, corresponding to a single-base accuracy of more than 99.99%. The final assembly contains 7 gaps, compared with 25 gaps in the previous draft assembly [7], indicating improved contiguity. A side-by-side comparison of the available assembly metrics is provided in Table S5. The quality of this assembly is greater than that of a previous study [52] in terms of total assembled size and gap count. For gene annotation, we predicted 21,404 protein-coding genes. This high-quality genome assembly represents a valuable genomic resource for the marine teleost lineage Triglidae and will facilitate future studies on marine fish adaptation and evolution.

**Figure 1:**
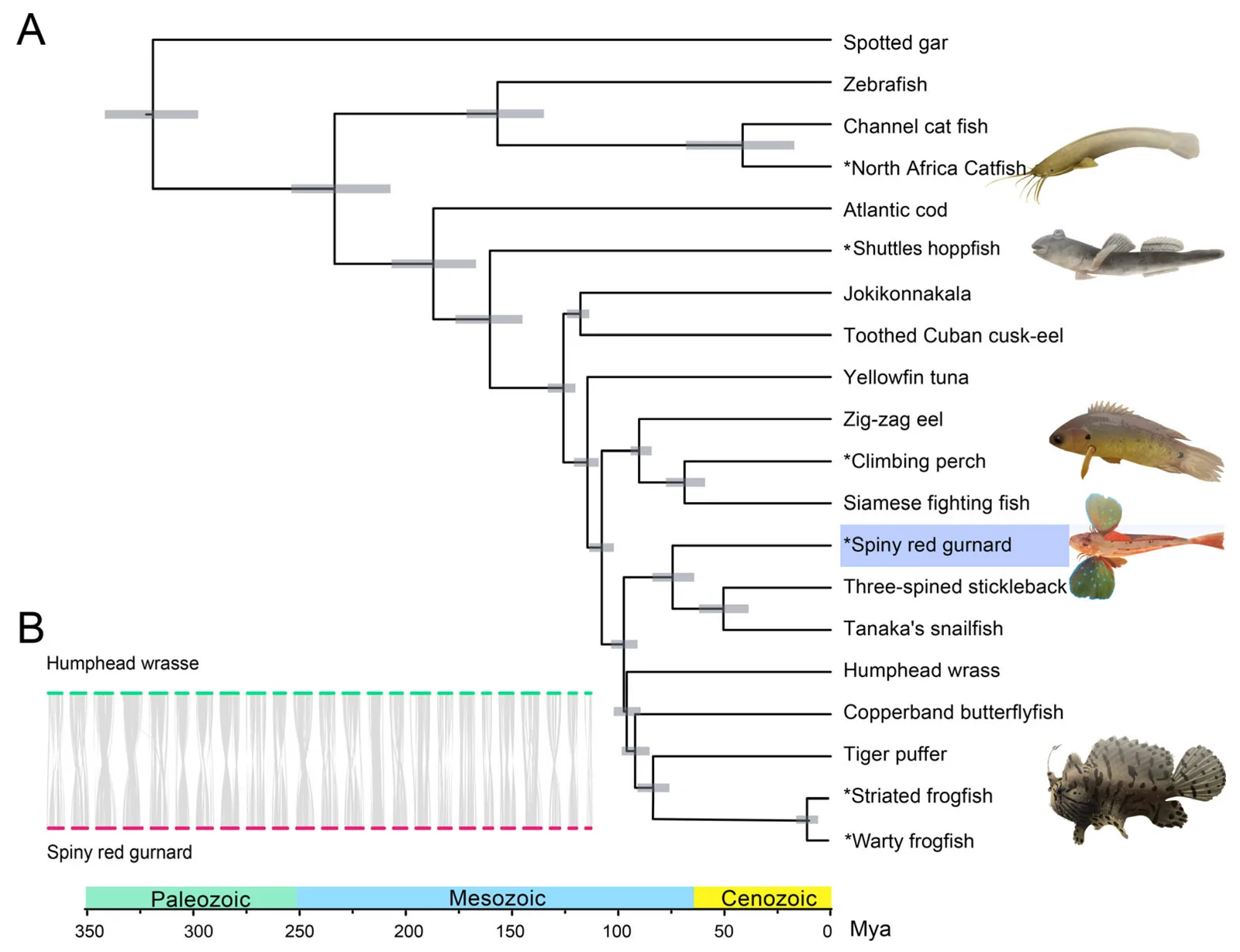
Phylogenomic relationships and chromosomal synteny of the spiny red gurnard and comparative fish lineages. (**A**) Whole-genome phylogeny covering five walking fish lineages (denoted by an asterisk). The genome newly assembled spiny red gurnard is highlighted by a blue ribbon. Gray bars at internal nodes indicate the 95% highest posterior density (HPD) intervals of estimated divergence times. (**B**) Chromosomal synteny between spiny red gurnard and the humphead wrasse.

Using the newly assembled genome together with publicly available genomes of the other four walking-associated fish lineages, we reconstructed a phylogenetic tree covering the five “walking” fish from Lepisosteiformes to Perciformes and identified the spiny red gurnard grouped with Perciformes (Figure 1A), which is consistent with previous phylogenetic studies that identified Triglidae within bony fishes based on molecular and genomic data [53]. Clearly identifiable chromosome segments were observed between the genome of the spiny red gurnard and the genome of Humphead wrasse, the representative species of Eupercaria, reflecting highly conserved synteny in Eupercaria (Figure 1B).

### 3.2. Genetic Signatures Associated with Walking-Related Locomotion

**Figure 2:**
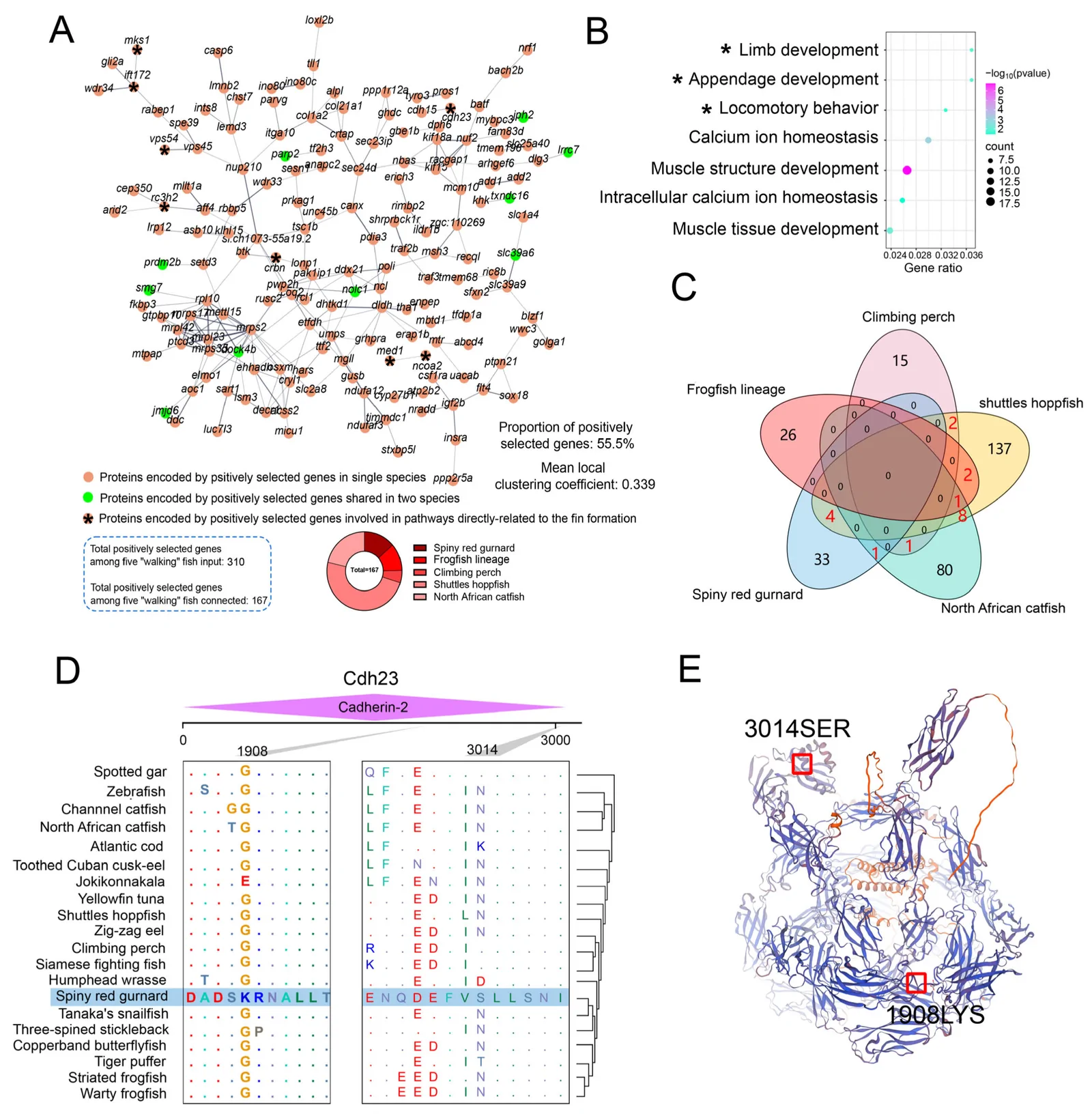
Genetic basis of evolution and organ adaptation in spiny red gurnard. (**A**) Protein–protein interaction network constructed by PSGs from five walking-associated fish lineages. The proportion of PSGs from each species in the network was shown in the bottom. The circle denoted by asterisk represents the encoded proteins involved in the pathways directly related to the fin formation shown in (**B**) by an asterisk. Line thickness indicates the strength of data support. The proportion of connected PSGs was calculated as the number of connected nodes (167) divided by the number of STRING-retained nodes (301). The network contains 142 edges. The mean local clustering coefficient (ranging from 0 to 1) measures the degree to which nodes in the network tend to cluster together; higher values indicate a greater tendency to form tightly connected modules. (**B**) Bubble plot of the fin-related pathways that consist of the genes from the protein network. The pathways directly related to the fin formation were shown by blue. Color scale represents adjusted *p* values. (**C**) Venn diagram of PSGs among five walking-associated fish lineages. The number of shared PSGs between species are highlighted by red. (**D**) Positively selected amino acid residues in protein encoded by positively selected *cdh23* gene in spiny red gurnard. (**E**) 3D structure of the Cdh23 protein. Two positively selected residues in spiny red gurnard are highlighted by the red frames.

To investigate the genetic basis for fish “walking” behavior, we first identified PSGs in the genomes of five walking-associated fish lineages. Given that similar locomotor functions may arise from candidate functional similarity at the PPI network level rather than from identical individual genes, we integrated these PSGs and constructed a PPI network using their encoded proteins. In total, we identified 330 PSGs, including 38 in spiny red gurnard, 154 in shuttles hoppfish, 29 in striated frogfish, 18 in climbing perch, and 91 in North African catfish (Table S3). For each lineage, the numbers of valid tests, nominal candidates (raw *p* < 0.05), and FDR-supported PSGs (BH-adjusted *p* < 0.05) were as follows: spiny red gurnard, 87 valid tests, 38 nominal candidates, 29 FDR-supported PSGs; striated frogfish, 54 valid tests, 30 nominal candidates, 25 FDR-supported PSGs; climbing perch, 34 valid tests, 18 nominal candidates, 14 FDR-supported PSGs; shuttles hoppfish, 551 valid tests, 154 nominal candidates, 92 FDR-supported PSGs; North African catfish, 346 valid tests, 91 nominal candidates, 56 FDR-supported PSGs. Of the 310 unique candidate PSGs submitted to STRING, 301 were retained after mapping. Among these, 167 nodes had at least one association (142 edges), whereas 134 remained isolated. The 167 connected nodes were used for subsequent enrichment analysis. Ten proteins (Parp2, Prdm2b, Smg7, Dock4b, Jmjd6, Nolc1, Slc39a6, Txndc16, Lrrc7, and Jph2) were shared among lineages (Figure 2A; highlighted in green). Additionally, eight PSG-encoded proteins (Mks1, Ift172, Vps54, Rc3h2, Crbn, Med1, Ncoa2, and Cdh23) were found to be closely related to fin formation (Figure 2A; highlighted with asterisks).

According to network theory, high clustering coefficients indicate that proteins tend to operate within tightly interconnected complexes rather than in isolation [54,55]. Compared with the networks constructed separately for each species (Figure S2), the PPI network contained a larger proportion of interacting proteins and had a mean local clustering coefficient of 0.339, which was lower only than that of the network constructed for the striated frogfish (0.357). These descriptive network properties show that a subset of the PSGs from different lineages participate in interconnected protein–protein interaction relationships. The network was therefore used as an exploratory framework to identify functional associations and biological processes shared among candidate genes across lineages. Subsequent Gene Ontology enrichment analysis of the 167 network proteins revealed annotations of biological processes related to appendage development and locomotion (Table S6), such as limb development, appendage development, and locomotory behavior (Figure 2A,B; denoted by asterisks). These annotations indicate conserved developmental and functional associations among candidate genes, but they do not independently demonstrate the evolution of walking fins. Such enrichment is consistent with, but does not demonstrate, a role in walking-fin evolution. We therefore describe these results as candidate functional associations that require further experimental validation. Such “functional convergence” is widespread. Echolocating mammals converge on hearing-related gene networks [56]. The stripe color pattern of cichlid fishes has undergone repeated evolution through regulatory changes at the *agrp2* locus [57]. Eye degeneration in cave fish involves the convergent loss of visual gene networks [58,59]. These examples illustrate that convergence occurs at multiple levels from individual genes to pathways to networks. Our findings extend this principle to locomotor adaptation, indicating functional association at the protein interaction level across independently evolved walking-associated lineages.

In addition to the functional association observed at the protein interaction network level, we identified multiple PSGs shared between specific walking-associated fish lineages, suggesting that convergent evolution may also occur at a finer, gene-level scale. For instance, the spiny red gurnard shares four PSGs (*txndc16*, *c6*, *nolc1*, *smg7*) with the shuttles hoppfish and one (*fut9a*) with the North African catfish; the North African catfish shares eight PSGs (*prdm2b*, *slc39a6*, *parp2*, *ap3b2*, *igsf8*, *jph2*, *lrrc7*, *dock4b*) with the shuttles hoppfish, one (*inpp5l*) with the climbing perch, and, together with the shuttles hoppfish and striated frogfish, shares *ccdc80*; the shuttles hoppfish also shares *jmjd6* and *mrps2* with the striated frogfish, and *zgc:172180* and *cherp* with the climbing perch. Among these genes, *jph2* is specifically expressed in the pectoral fin musculature of zebrafish (ZFIN: ZDB-GENE-030131-9848). Junctophilin-2, the protein it encodes, is a key component of excitation–contraction coupling in skeletal muscle and directly participates in muscle contraction, providing the mechanical force required for “walking” [60,61]. *ccdc80* (whose zebrafish homolog is *ccdc80-l1*) plays an essential role in motoneuron axon guidance; its loss of function leads to disorganized migration of primary and secondary motoneurons and abnormal embryonic swimming behavior, without affecting muscle development itself [62]. Notably, *ccdc80* showed clear expression in several fin-associated cell populations, with the highest expression detected in fibroblasts and osteoblast 1 cells, and lower expression in glial cells, osteoblast 2 cells, and endothelial cells (Figure S3). This expression pattern provides tissue- and cell-type-level support that *ccdc80* is expressed within the specialized pectoral-fin system, including cell populations associated with connective tissue and skeletal components. *lrrc7* encodes Densin-180, a core scaffolding protein of the postsynaptic density, which is critical for maintaining normal synaptic spine structure and function and is involved in synaptic plasticity [63] and skeletal muscle function [64]. *ap3b2* encodes the β2 subunit of the neuron-specific AP-3 complex, which is responsible for the intracellular transport of synaptic vesicles; mouse knockout experiments have confirmed that its deletion alters the protein composition of synaptic vesicles and leads to abnormal neurotransmitter release [65]. The recurrent appearance of these genes in independently evolved walking-associated lineages, together with their reported functions in neural signaling (*lrrc7*, *ap3b2*), muscle contraction (*jph2*), and locomotor-related processes (*ccdc80*), identifies them as candidate genes potentially related to neuromuscular function. The pectoral-fin expression of *ccdc80* further supports its relevance to this tissue, although the specific contributions of these genes to walking-associated locomotion remain to be experimentally established. Their parallel selection may contribute to candidate functional similarity associated with walking locomotion.

Unlike the above-mentioned shared PSGs, *cdh23* (FDR = 0.194) was detected as a PSG only in the spiny red gurnard and not in the other four walking-associated fish lineages. From the protein site perspective, two sites under positive selection (posterior probability > 0.9) were located in the α-helix and the random coil of Cdh23 (Figure 2D,E). Two candidate positively selected substitutions, G1908K and N3014S, were mapped onto the predicted Cdh23 structure. These substitutions occur in distinct structural regions and involve changes in residue physicochemical properties, suggesting that they may alter the local structural environment of Cdh23.

The residue 3014 in the α-helix shows an asparagine to serine substitution (N3014S), whereas residue 1908 in the random-coil region shows a glycine-to-lysine substitution (G1908K). These substitutions involve changes in residue physicochemical properties and may alter the local structural environment of Cdh23 (Table S7). Cdh23 has well-established dual roles in vertebrates: in zebrafish, it is essential for mechanotransduction in hair cells of the inner ear [66]; in humans, mutations in this gene cause Usher syndrome type 1D (deafness and vestibular dysfunction) [67] and increases the risk of semicircular canal dehiscence [68]; and in a recent mouse model, Cdh23 mutation leads to abnormal stereocilia bundle development, altered otolith morphology and activation of the p53/FoxO pathway [69]. Cdh23 has established roles in mechanotransduction and sensory function in vertebrate systems, but these functions do not directly demonstrate a role in tactile sensing or mechanical support of the free pectoral fin rays in the spiny red gurnard. The lineage-specific positive-selection signal and amino-acid changes identified here therefore nominate *cdh23* as a candidate potentially related to mechanosensory function, although its physiological role in the pectoral fin remains to be experimentally validated. These observations complement our previous identification of the chemosensory receptor *srgor* in the same appendage [7].

The evolution of walking-associated locomotor systems involves a multi-level genetic architecture driven by candidate functional similarity at the protein interaction network level, parallel selection of shared genes, and lineage-specific molecular innovations (such as *cdh23* in the spiny red gurnard). These findings provide new insights into the molecular mechanisms of complex locomotor adaptations in fishes and highlight the importance of integrating both coding and non-coding, conserved and lineage-specific changes in studies of convergent evolution.

### 3.3. Regulatory Evolution of Fin-Development-Associated CNEs

**Figure 3:**
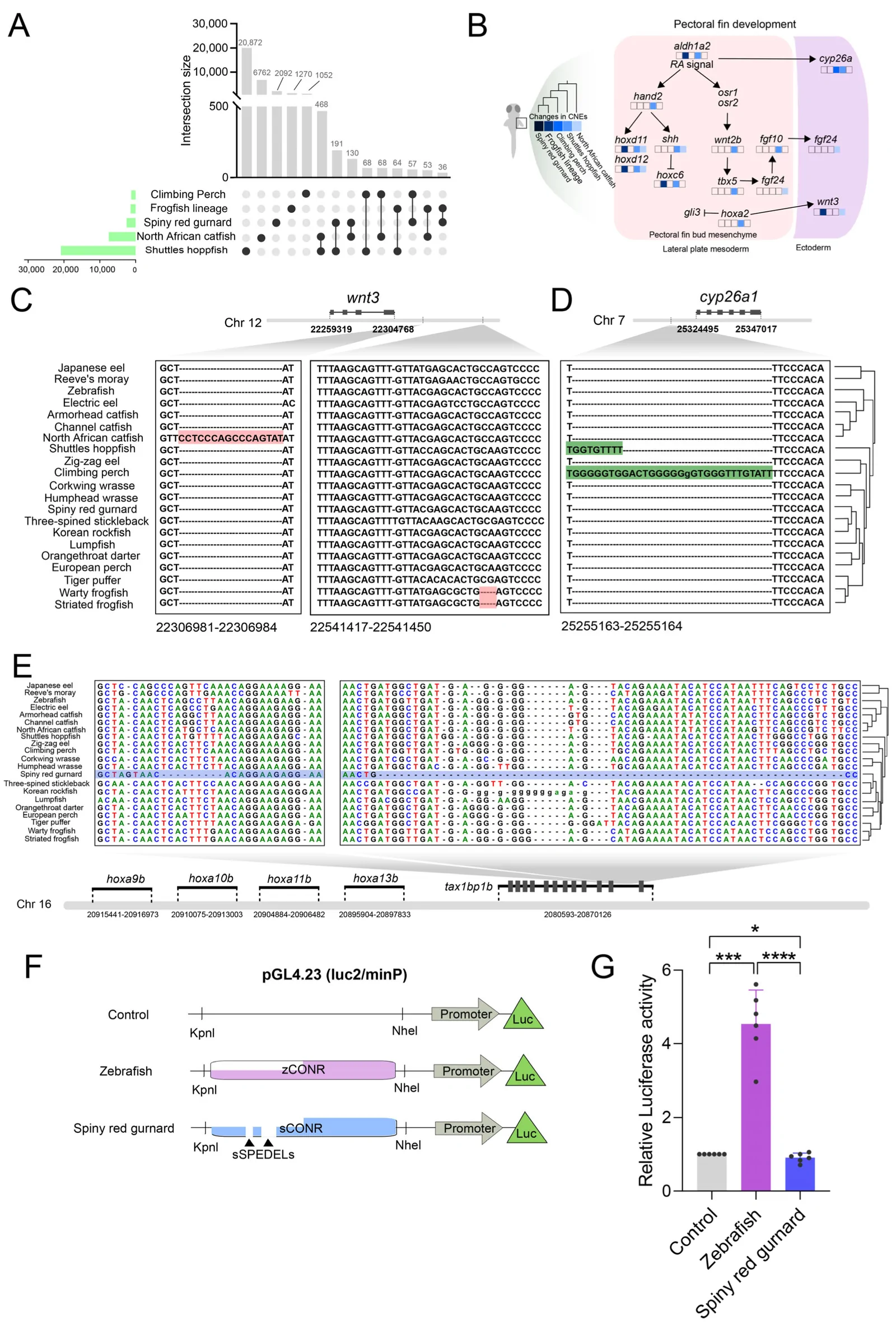
Regulatory evolution of fin-development CNEs in walking-associated fish lineages. (**A**) UpSet plot of unique and shared changes in CNEs identified in five walking-associated fish lineages. (**B**) The pathways linking pectoral fin development consist of genes and their possible regulatory CNEs. Different colors or the absence of color in squares indicate the presence or the absence of CNEs in different walking-associated fish lineages. RA (retinoic acid signaling). *Hoxa2* is included based on documented expression in pectoral fin buds, although its functional role remains unclear. (**C**) Partial sequence alignment of CNEs located on the adjacent area of *wnt3* (Chr12: 22306981–22306984 and 22541417–22541450). (**D**) Partial sequence alignment of CNEs near *cyp26a1* (Chr7: 25255163–25255164) involved in the pectoral fin-related pathways. Pink and green represent the changes in sites within CNEs between walking-associated fish lineages. (**E**) A spiny red gurnard-specific CNE deletion located within an intron of *tax1bp1b*. The orthologous zebrafish region on chromosome 16 is shown together with the neighboring hoxab cluster genes (*hoxa9b*, *hoxa10b*, *hoxa11b*, and *hoxa13b*) to illustrate their genomic context. (**F**) Vector design for dual-luciferase reporter assay. sSPEDELs denote spiny red gurnard-specific CNE deletions, sCONR, the conserved non-coding region of the spiny red gurnard; zCONR, the conserved non-coding region of zebrafish. (**G**) Relative Luciferase activity activated by different vectors. Statistical significance was evaluated using unpaired two-tailed Student’s *t*-tests with Bonferroni correction for multiple comparisons. * *p* < 0.05; *** *p* < 0.001; **** *p* < 0.0001. Highlighted differences are located near the same gene but occur in different sequence blocks; they do not necessarily represent independent changes to the same orthologous regulatory element. Gene names and coordinates were verified against the zebrafish GRCz11 annotation.

CNEs are regulatory sequences that control the expression of genes involved in development [70,71]; changes in CNEs can alter gene expression patterns without modifying protein sequences and have been repeatedly linked to morphological evolution [18]. To investigate regulatory changes associated with fin-related developmental evolution across walking-associated lineages, we identified CNEs adjacent to appendage development genes across the five focal species. The number of species-specific CNE changes ranged from approximately 1052 to 6762 per species, with shuttles hoppfish showing a notably high count of 20,872 (Figure 3A). When CNE-associated genes were mapped to fin development processes, we observed that certain developmental pathways, such as the aldh1a2–hand2–hoxd11/12–shh–hoxc6 axis, contained shared CNEs in at least two species, whereas others, including the aldh1a2–osr1/2 axis and ectoderm-associated genes, showed mostly species-specific CNEs (Figure 3B). Although hoxa2 expression in pectoral fin buds has been reported in zebrafish and in the anterior pectoral fin of skate [72,73], its functional role in pectoral fin development remains unclear. We therefore include hoxa2 here only as a pectoral-fin-associated gene based on its documented expression pattern, without implying a defined functional role in this process. Because CNE–gene assignments are based on genomic proximity, these pathway-level associations remain uncertain and should be considered hypothesis-generating. These results indicate that most CNE changes are lineage-specific, yet different developmental modules exhibit varying degrees of regulatory conservation. Lineage-specific callable CNE denominators were not calculated; therefore, CNE counts should be interpreted as candidate changes rather than normalized estimates of regulatory innovation.

The predominant lineage-specific pattern of CNE changes suggests that different lineages may use divergent regulatory mechanisms to achieve their specialized fin morphologies. However, we note that developmental system drift (DSD) sensu stricto refers to divergence in developmental mechanisms while the phenotype remains conserved [74]. In our case, the “walking” phenotype is thought to have evolved independently in multiple lineages; therefore, the lineage-specific regulatory changes are better interpreted as independent molecular routes to a convergent phenotype, rather than as evidence of DSD. We therefore describe these patterns as lineage-specific regulatory evolution associated with independent origins of walking-associated morphology. Interestingly, the observation that certain core developmental pathways (e.g., the hand2–hox axis) contain shared CNEs across multiple species suggests that while most regulatory evolution is lineage-specific, core developmental modules may be preferentially preserved or repeatedly targeted during the evolution of walking-associated locomotor systems.

Despite this overall pattern of lineage-specificity, we identified several cases of shared CNE changes in specific species pairs. CNE changes near *wnt3* were detected in both striated frogfish and North African catfish, and CNE changes near *cyp26a1* were detected in shuttles hoppfish and climbing perch (Figure 3C,D). However, because these changes occur in different sequence blocks within the same genomic region, we cannot determine whether they affect the same orthologous regulatory element. We therefore refer to them as candidate convergent changes at the locus level (i.e., CNE changes near the same gene), rather than claiming element-level or nucleotide-level convergence. *Wnt3* maintains the apical ectodermal ridge during pectoral fin and limb development [73,75]. *Cyp26a1* locally degrades retinoic acid (RA), a key morphogen that patterns the anterior–posterior axis of developing appendages; by shaping RA gradients, Cyp26a1 helps establish proper proximodistal patterning in fins and limbs [76,77]. Modifications in *cyp26a1* regulatory elements could therefore alter RA signaling dynamics during fin development, leading to changes in skeletal patterning. The convergent changes observed in these two amphibious fishes may represent a common molecular solution for producing fins adapted to weight-bearing terrestrial locomotion, which is consistent with the conserved role of RA signaling in appendage patterning across vertebrates [78].

Regulatory changes in non-coding elements are increasingly recognized as important drivers of morphological evolution. Notably, we discovered a spiny red gurnard-specific deletion of a CNE within an intron of the *tax1bp1b* gene (Figure 3E), a gene located in a genomic region previously shown to contain regulatory elements important for fin development. This candidate deletion was supported by read-level evidence and assembly continuity (Figure 3E). Genomic localization analysis further revealed that this intronic CNE is near multiple pectoral fin-related *hox* genes (Figure 3E). Ahn & Ho [79] demonstrated that multiple genes (including *tax1bp1b*) adjacent to the zebrafish *hox* cluster are located within a shared regulatory landscape that influences gene expression in the distal fin mesenchyme. Furthermore, the CNE is near multiple *hoxab* cluster genes (corresponding to human HOXA) (*hoxa9b*, *hoxa10b*, *hoxa11b*, and *hoxa13b*; Figure 3E). HoxA cluster genes are among the main regulatory factors along the proximal-distal and anterior–posterior axes of appendages [80,81]. However, these studies provide general background and do not directly demonstrate that this specific CNE regulates the neighboring HoxA genes.

To assess the regulatory potential of this candidate CNE in vitro, we constructed three dual-luciferase reporter vectors (Figure 3F): an empty vector control, a vector containing the zebrafish conserved non-coding region (zCONR), and a vector containing the spiny red gurnard conserved non-coding region (sCONR). The sCONR sequence naturally contains a species-specific CNE deletion. Dual-luciferase reporter assays showed that the zCONR construct drove luciferase expression significantly above that of the empty vector control, consistent with enhancer-like activity in the HEK293T reporter system. In contrast, the sCONR construct showed significantly reduced reporter activity relative to the zCONR construct and was also lower than the empty-vector control, suggesting a preliminary difference in heterologous reporter activity; the endogenous target and in vivo function of this CNE remain unknown in the spiny red gurnard lineage (Figure 3G). More broadly, regulatory sequence changes have been associated with morphological evolution in fishes, including deletion of the *pitx1* enhancer associated with pelvic reduction in three-spined stickleback [20,82], and the loss of function of enhancers near the cbs gene during eye degeneration in cave fish [83].

Although direct evidence that this CNE directly regulates *tax1bp1b* or adjacent *Hox* genes is lacking, and this proximity-based association remains hypothetical, the differential reporter activity observed in HEK293T cells suggests altered regulatory potential between the zebrafish and spiny red gurnard sequences. However, these results do not establish the endogenous target gene(s) or an in vivo role of this CNE in pectoral-fin development. Further functional experiments, such as transgenic reporter assays or genome editing in an appropriate fish model, are needed to determine its in vivo regulatory activity and potential effects on fin morphology. Taken together, this lineage-specific CNE alteration represents a candidate regulatory change that may be associated with fin morphological diversification, highlighting the importance of exploring non-coding regions in studies of adaptive evolution.

The association between this CNE and tax1bp1b or the neighboring Hox genes is based on genomic proximity and remains hypothetical; further experimental validation is required. The differential reporter activity observed in HEK293T cells indicates altered regulatory potential between the zebrafish and spiny red gurnard sequences, but does not establish an in vivo regulatory relationship.

## 4. Conclusions

This study suggests a dual pattern of genomic variation, comprising shared functional associations among candidate genes and predominantly lineage-specific regulatory changes. The improved genome assembly and annotation data provided here will serve as a valuable reference for future studies on pectoral fin development, convergent evolution, and adaptive locomotion in teleosts. At the regulatory level, we observed predominantly lineage-specific changes reflecting unique evolutionary trajectories, occasionally accompanied by candidate convergent changes near *wnt3* and *cyp26a1*. At the protein interaction level, PSGs from different lineages showed functional associations within a shared interaction network enriched for processes related to fin development and locomotion. This integrative analysis provides an exploratory view of the genetic architecture underlying this remarkable adaptation. Future studies should focus on functionally validating the regulatory elements identified here, using transgenic approaches in model organisms such as zebrafish to evaluate the enhancer activity of specific CNEs and assess the phenotypic consequences of their modification. Additionally, to better understand the regulatory evolution associated with walking locomotion, comparative epigenomic analyses across multiple walking-associated fish lineages should be conducted. Examining chromatin accessibility and histone modifications can reveal regulatory information beyond DNA sequence variation. This approach helps elucidate how key regulatory elements—such as enhancers—are activated or repressed during evolution, ultimately providing deeper insights into the dynamic regulatory landscape underlying fin morphological diversification.

## Data Availability

The raw sequencing data (PacBio HiFi, Hi-C, and Illumina short reads) are from our previous study [7] and have been deposited in the NCBI Sequence Read Archive (SRA) under BioProject accession number PRJNA1154553 (SRR30484483, SRR30484484, SRR30484485). The final genome assembly (FASTA) and gene annotation files (GFF) are available via Figshare at https://doi.org/10.6084/m9.figshare.32815106.

## Supplementary Materials

The following supporting information can be downloaded at https://www.mdpi.com/article/10.3390/biology15211990/s1. Table S1. Phenotype matrix of the five focal walking-associated fish species; Table S2. Summary of species and genome assemblies included in this study; Table S3. Positively selected genes (*p* value < 0.05) identified from five walking-asscoiated fish species; Table S4. Inserts and primers used in the dual-luciferase reporter assay; Table S5. Comparison of the previous and current genome assemblies of the spiny red gurnard; Table S6. Gene ontology-Biological processes enriched by five species PSGs; Table S7. Domain annotation of Cdh23; Figure S1. Genomic landscape of the spiny red gurnard (*Chelidonichthys spinosus*). Circos plot illustrating the distribution of various genomic features across the 24 chromosomes of the spiny red gurnard. The tracks from outside to inside represent chromosomes (1–24), gene density, DNA transposons, tandem repeats (TR), LTR retrotransposons, SINEs, LINEs, and GC content, respectively. The color scale for the inner tracks ranges from minimum (Min, yellow/light blue) to maximum (Max, dark blue) density values; Figure S2. Species-specific protein-protein interaction (PPI) networks of positively selected genes (PSGs). PPI networks were constructed for the individual walking-associated lineages based on their respective PSGs. (A) Shuttles hoppfish; (B) Spiny red gurnard; (C) North African catfish; (D) Striated frogfish. The climbing perch is not shown because its PSG number was insufficient to construct a meaningful network. The proportion of positively selected genes mapped to the network and the mean local clustering coefficient are indicated below each panel. Figure S3. Single-cell expression profile of *ccdc80* across distinct cell types in the pectoral fin. Bar plot showing the average expression levels of *ccdc80* across various cell populations identified from the single-cell RNA-seq dataset of the spiny red gurnard pectoral fin. The highest expression is observed in fibroblasts and “osteoblast 1” cells.

## References

[1] Gould S.J., Vrba E.S. (1982). Exaptation—A Missing Term in the Science of Form. Paleobiology.

[2] Webb P.W. (1984). Body Form, Locomotion and Foraging in Aquatic Vertebrates. Integr. Comp. Biol..

[3] Tanaka M. (2016). Developmental Mechanism of Limb Field Specification along the Anterior-Posterior Axis during Vertebrate Evolution. J. Dev. Biol..

[4] Newton A.H., Smith C.A. (2021). Regulation of Vertebrate Forelimb Development and Wing Reduction in the Flightless Emu. Dev. Dyn..

[5] Allard C.A., Herbert A.L., Krueger S.P., Liang Q., Walsh B.L., Rhyne A.L., Gourlay A.N., Seminara A., Baldwin M.W., Kingsley D.M. (2024). Evolution of Novel Sensory Organs in Fish with Legs. Curr. Biol..

[6] Silver W.L., Finger T.E. (1984). Electrophysiological Examination of a Non-Olfactory, Non-Gustatory Chemosense in the Searobin, Prionotus Carolinus. J. Comp. Physiol. A.

[7] Li L., Fan D., Zhu C., Liu Z., Huang W., Xin P., Yue H., Li M., Wang Y., Xu W. (2025). Exaptation of Pectoral Fins for Olfaction in the Spiny Red Gurnard (*Chelidonichthys spinosus*) through an Ancient Receptor. Sci. China Life Sci..

[8] Herbert A.L., Allard C.A.H., McCoy M.J., Wucherpfennig J.I., Krueger S.P., Chen H.I., Gourlay A.N., Jackson K.D., Abbo L.A., Bennett S.H. (2024). Ancient Developmental Genes Underlie Evolutionary Novelties in Walking Fish. Curr. Biol..

[9] Petersen J.C., Ramsay J.B. (2020). Walking on Chains: The Morphology and Mechanics behind the Fin Ray Derived Limbs of Sea-Robins. J. Exp. Biol..

[10] King H.M., Shubin N.H., Coates M.I., Hale M.E. (2011). Behavioral Evidence for the Evolution of Walking and Bounding before Terrestriality in Sarcopterygian Fishes. Proc. Natl. Acad. Sci. USA.

[11] Macesic L.J., Kajiura S.M. (2010). Comparative Punting Kinematics and Pelvic Fin Musculature of Benthic Batoids. J. Morphol..

[12] Yang Y., Yoo J.Y., Baek S.H., Song H.Y., Jo S., Jung S.H., Choi J.H. (2022). Chromosome-Level Genome Assembly of the Shuttles Hoppfish, Periophthalmus Modestus. Gigascience.

[13] Hu M., Fan D., Hao J., Zhang F., Xu W., Zhu C., Wang K., Song X., Li L. (2024). A Chromosome-Level Genome of the Striated Frogfish (*Antennarius striatus*). Sci. Data.

[14] Kuznetsov A.N. (2022). How Big Can a Walking Fish Be? A Theoretical Inference Based on Observations on Four Land-Dwelling Fish Genera of South Vietnam. Integr. Zool..

[15] Pace C.M., Gibb A.C. (2014). Sustained Periodic Terrestrial Locomotion in Air-Breathing Fishes. J. Fish. Biol..

[16] Sackton T.B., Clark N. (2019). Convergent Evolution in the Genomics Era: New Insights and Directions. Philos. Trans. R. Soc. B Biol. Sci..

[17] Losos J.B. (2011). Convergence, Adaptation, and Constraint. Evolution.

[18] Carroll S.B. (2008). Evo-Devo and an Expanding Evolutionary Synthesis: A Genetic Theory of Morphological Evolution. Cell.

[19] Irizarry J., Stathopoulos A. (2021). Dynamic Patterning by Morphogens Illuminated by Cis-Regulatory Studies. Development.

[20] Chen H.I., Turakhia Y., Bejerano G., Kingsley D.M. (2023). Whole-Genome Comparisons Identify Repeated Regulatory Changes Underlying Convergent Appendage Evolution in Diverse Fish Lineages. Mol. Biol. Evol..

[21] Chen S., Zhou Y., Chen Y., Gu J. (2018). Fastp: An Ultra-Fast All-in-One FASTQ Preprocessor. Bioinformatics.

[22] Luo R., Liu B., Xie Y., Li Z., Huang W., Yuan J., He G., Chen Y., Pan Q., Liu Y. (2015). Erratum: SOAPdenovo2: An Empirically Improved Memory-Efficient Short-Read de Novo Assembler. Gigascience.

[23] Cheng H., Concepcion G.T., Feng X., Zhang H., Li H. (2021). Haplotype-Resolved de Novo Assembly Using Phased Assembly Graphs with Hifiasm. Nat. Methods.

[24] Durand N.C., Shamim M.S., Machol I., Rao S.S.P., Huntley M.H., Lander E.S., Aiden E.L. (2016). Juicer Provides a One-Click System for Analyzing Loop-Resolution Hi-C Experiments. Cell Syst..

[25] Dudchenko O., Batra S.S., Omer A.D., Nyquist S.K., Hoeger M., Durand N.C., Shamim M.S., Machol I., Lander E.S., Aiden A.P. (2017). De Novo Assembly of the Aedes Aegypti Genome Using Hi-C Yields Chromosome-Length Scaffolds. Science.

[26] Xu M., Guo L., Gu S., Wang O., Zhang R., Peters B.A., Fan G., Liu X., Xu X., Deng L. (2020). TGS-GapCloser: A Fast and Accurate Gap Closer for Large Genomes with Low Coverage of Error-Prone Long Reads. Gigascience.

[27] Manni M., Berkeley M.R., Seppey M., Zdobnov E.M. (2021). BUSCO: Assessing Genomic Data Quality and Beyond. Curr. Protoc..

[28] Rhie A., Walenz B.P., Koren S., Phillippy A.M. (2020). Merqury: Reference-Free Quality, Completeness, and Phasing Assessment for Genome Assemblies. Genome Biol..

[29] Saha S., Bridges S., Magbanua Z.V., Peterson D.G. (2008). Empirical Comparison of Ab Initio Repeat Finding Programs. Nucleic Acids Res..

[30] Tarailo-Graovac M., Chen N. (2009). Using RepeatMasker to Identify Repetitive Elements in Genomic Sequences. Curr. Protoc. Bioinform..

[31] Stanke M., Keller O., Gunduz I., Hayes A., Waack S., Morgenstern B. (2006). AUGUSTUS: A b Initio Prediction of Alternative Transcripts. Nucleic Acids Res..

[32] Zhang P., Xu T., Wang S., Yang X., Sun P., Jia P., Wang B., Bush S., Ning Z., Ye K. (2026). Highly Accurate Ab Initio Gene Annotation with ANNEVO. Nat. Methods.

[33] Bushmanova E., Antipov D., Lapidus A., Prjibelski A.D. (2019). RnaSPAdes: A de Novo Transcriptome Assembler and Its Application to RNA-Seq Data. Gigascience.

[34] Haas B., Papanicolaou A., Yassour M. (2017). TransDecoder. https://github.com/TransDecoder/TransDecoder.

[35] Li H. (2023). Protein-to-Genome Alignment with Miniprot. Bioinformatics.

[36] Haas B.J., Salzberg S.L., Zhu W., Pertea M., Allen J.E., Orvis J., White O., Robin C.R., Wortman J.R. (2008). Automated Eukaryotic Gene Structure Annotation Using EVidenceModeler and the Program to Assemble Spliced Alignments. Genome Biol..

[37] Buchfink B., Xie C., Huson D.H. (2014). Fast and Sensitive Protein Alignment Using DIAMOND. Nat. Methods.

[38] Katoh K., Standley D.M. (2013). MAFFT Multiple Sequence Alignment Software Version 7: Improvements in Performance and Usability. Mol. Biol. Evol..

[39] Nguyen L.T., Schmidt H.A., Von Haeseler A., Minh B.Q. (2015). IQ-TREE: A Fast and Effective Stochastic Algorithm for Estimating Maximum-Likelihood Phylogenies. Mol. Biol. Evol..

[40] Zhang C., Rabiee M., Sayyari E., Mirarab S. (2018). ASTRAL-III: Polynomial Time Species Tree Reconstruction from Partially Resolved Gene Trees. BMC Bioinform..

[41] Yang Z. (2007). PAML 4: Phylogenetic Analysis by Maximum Likelihood. Mol. Biol. Evol..

[42] Kumar S., Stecher G., Suleski M., Blair Hedges S. (2017). TimeTree: A Resource for Timelines, Timetrees, and Divergence Times. Mol. Biol. Evol..

[43] Tang H., Bowers J.E., Wang X., Ming R., Alam M., Paterson A.H. (2008). Synteny and Collinearity in Plant Genomes. Science.

[44] Abramson J., Adler J., Dunger J., Evans R., Green T., Pritzel A., Ronneberger O., Willmore L., Ballard A.J., Bambrick J. (2024). Accurate Structure Prediction of Biomolecular Interactions with AlphaFold 3. Nature.

[45] Kiełbasa S.M., Wan R., Sato K., Horton P., Frith M.C. (2011). Adaptive Seeds Tame Genomic Sequence Comparison. Genome Res..

[46] Blanchette M., Kent W.J., Riemer C., Elnitski L., Smith A.F.A., Roskin K.M., Baertsch R., Rosenbloom K., Clawson H., Green E.D. (2004). Aligning Multiple Genomic Sequences with the Threaded Blockset Aligner. Genome Res..

[47] Hubisz M.J., Pollard K.S., Siepel A. (2011). PHAST and RPHAST: Phylogenetic Analysis with Space/Time Models. Brief. Bioinform..

[48] Siepel A., Bejerano G., Pedersen J.S., Hinrichs A.S., Hou M., Rosenbloom K., Clawson H., Spieth J., Hillier L.D.W., Richards S. (2005). Evolutionarily Conserved Elements in Vertebrate, Insect, Worm, and Yeast Genomes. Genome Res..

[49] Zhu C., Li S., Zhang D., Zhang J., Wang G., Zhou B., Zheng J., Xu W., Wang Z., Gao X. (2024). Convergent Degenerated Regulatory Elements Associated with Limb Loss in Limbless Amphibians and Reptiles. Mol. Biol. Evol..

[50] Szklarczyk D., Kirsch R., Koutrouli M., Nastou K., Mehryary F., Hachilif R., Gable A.L., Fang T., Doncheva N.T., Pyysalo S. (2023). The STRING Database in 2023: Protein-Protein Association Networks and Functional Enrichment Analyses for Any Sequenced Genome of Interest. Nucleic Acids Res..

[51] Kolberg L., Raudvere U., Kuzmin I., Adler P., Vilo J., Peterson H. (2023). G:Profiler-Interoperable Web Service for Functional Enrichment Analysis and Gene Identifier Mapping (2023 Update). Nucleic Acids Res..

[52] Wang Y., Zhang H., Xian W., Iwasaki W. (2023). Chromosome Genome Assembly and Annotation of the Spiny Red Gurnard (*Chelidonichthys spinosus*). Sci. Data.

[53] Betancur R.R., Wiley E.O., Arratia G., Acero A., Bailly N., Miya M., Lecointre G., Ortí G. (2017). Phylogenetic Classification of Bony Fishes. BMC Evol. Biol..

[54] Watts D.J., Strogatz S.H. (1998). Collective Dynamics of ‘Small-World’ Networks. Nature.

[55] Barabási A.L., Oltvai Z.N. (2004). Network Biology: Understanding the Cell’s Functional Organization. Nat. Rev. Genet..

[56] Parker J., Tsagkogeorga G., Cotton J.A., Liu Y., Provero P., Stupka E., Rossiter S.J. (2013). Genome-Wide Signatures of Convergent Evolution in Echolocating Mammals. Nature.

[57] Kratochwil C.F., Liang Y., Gerwin J., Woltering J.M., Urban S., Henning F., Machado-Schiaffino G., Hulsey C.D., Meyer A. (2018). Agouti-Related Peptide 2 Facilitates Convergent Evolution of Stripe Patterns across Cichlid Fish Radiations. Science.

[58] Swaminathan A., Xia F., Rohner N. (2024). From Darkness to Discovery: Evolutionary, Adaptive, and Translational Genetic Insights from Cavefish. Trends Genet..

[59] Yang J., Chen X., Bai J., Fang D., Qiu Y., Jiang W., Yuan H., Bian C., Lu J., He S. (2016). The Sinocyclocheilus Cavefish Genome Provides Insaights into Cave Adaptation. BMC Biol..

[60] Perni S., Lavorato M., Beam K.G. (2017). De Novo Reconstitution Reveals the Proteins Required for Skeletal Muscle Voltage-Induced Ca2+ Release. Proc. Natl. Acad. Sci. USA.

[61] Garbino A., Van Oort R.J., Dixit S.S., Landstrom A.P., Ackerman M.J., Wehrens X.H.T. (2009). Molecular Evolution of the Junctophilin Gene Family. Physiol. Genom..

[62] Brusegan C., Pistocchi A., Frassine A., della Noce I., Schepis F., Cotelli F. (2012). Ccdc80-L1 Is Involved in Axon Pathfinding of Zebrafish Motoneurons. PLoS ONE.

[63] Willim J., Woike D., Greene D., Das S., Pfeifer K., Yuan W., Lindsey A., Itani O., Böhme A.L., Tibbe D. (2024). Variants in LRRC7 Lead to Intellectual Disability, Autism, Aggression and Abnormal Eating Behaviors. Nat. Commun..

[64] Espinosa Alvare K.G. (2020). Elucidating the Role of SPEG in Muscle Development and Function. Master’s Thesis.

[65] Newell-Litwa K., Salazar G., Smith Y., Faundez V. (2009). Roles of BLOC-1 and Adaptor Protein-3 Complexes in Cargo Sorting to Synaptic Vesicles. Mol. Biol. Cell.

[66] Söllner C., Rauch G.J., Siemens J., Geisler R., Schuster S.C., Müller U., Nicolson T. (2004). Mutations in Cadherin 23 Affect Tip Links in Zebrafish Sensory Hair Cells. Nature.

[67] Bolz H., Von Brederlow B., Ramírez A., Bryda E.C., Kutsche K., Nothwang H.G., Seeliger M., Cabrera M.d.C.-S., Vila M.C., Molina O.P. (2001). Mutation of CDH23, Encoding a New Member of the Cadherin Gene Family, Causes Usher Syndrome Type 1D. Nat. Genet..

[68] Noonan K.Y., Russo J., Shen J., Rehm H., Halbach S., Hopp E., Noon S., Hoover J., Eskey C., Saunders J.E. (2016). CDH23 Related Hearing Loss: A New Genetic Risk Factor for Semicircular Canal Dehiscence?. Otol. Neurotol..

[69] Tang L., Wu Y., Zhang K., Xie D., Kuang X., Wang L., Sun Z., Geng R., Hu J., Sun Y. (2025). Cdh23 Gene Mutation–Induced Vestibular Dysfunction in Mice: Abnormal Stereocilia Bundle and Otolith Development and Activation of P53/FoxO Signaling Pathway. J. Mol. Neurosci..

[70] Woolfe A., Goodson M., Goode D.K., Snell P., McEwen G.K., Vavouri T., Smith S.F., North P., Callaway H., Kelly K. (2005). Highly Conserved Non-Coding Sequences Are Associated with Vertebrate Development. PLoS Biol..

[71] Pennacchio L.A., Ahituv N., Moses A.M., Prabhakar S., Nobrega M.A., Shoukry M., Minovitsky S., Dubchak I., Holt A., Lewis K.D. (2006). In Vivo Enhancer Analysis of Human Conserved Non-Coding Sequences. Nature.

[72] Muto A., Ikeda S., Lopez-Burks M.E., Kikuchi Y., Calof A.L., Lander A.D., Schilling T.F. (2014). Nipbl and Mediator Cooperatively Regulate Gene Expression to Control Limb Development. PLoS Genet..

[73] Marlétaz F., de la Calle-Mustienes E., Acemel R.D., Paliou C., Naranjo S., Martínez-García P.M., Cases I., Sleight V.A., Hirschberger C., Marcet-Houben M. (2023). The Little Skate Genome and the Evolutionary Emergence of Wing-like Fins. Nature.

[74] True J.R., Haag E.S. (2001). Developmental System Drift and Flexibility in Evolutionary Trajectories. Evol. Dev..

[75] Nakamura T., Klomp J., Pieretti J., Schneider I., Gehrke A.R., Shubin A.N.H. (2015). Molecular Mechanisms Underlying the Exceptional Adaptations of Batoid Fins. Proc. Natl. Acad. Sci. USA.

[76] Naylor R.W., Skvarca L.B., Thisse C., Thisse B., Hukriede N.A., Davidson A.J. (2016). BMP and Retinoic Acid Regulate Anterior-Posterior Patterning of the Non-Axial Mesoderm across the Dorsal-Ventral Axis. Nat. Commun..

[77] Duarte-Olivenza C., Moran G., Hurle J.M., Montero J.A., Lorda-Diez C.I. (2025). Retinoic Acid Regulates the Proliferation, Differentiation, and Cell Death of Limb Skeletal Progenitors, Contributing to Establish the Size and Identity of the Digits. EvoDevo.

[78] Khan S., Ahmad K., Liu X., Liang Y. (2025). Unlocking the Potential of Retinoic Acid: A Comprehensive Review of Its Regulatory Role in Epimorphic Regeneration in Axolotl Limbs for Regenerative Medicine. Regen. Eng. Transl. Med..

[79] Ahn D., Ho R.K. (2008). Tri-Phasic Expression of Posterior Hox Genes during Development of Pectoral Fins in Zebrafish: Implications for the Evolution of Vertebrate Paired Appendages. Dev. Biol..

[80] Cass A.N., Elias A., Fudala M.L., Knick B.D., Davis M.C. (2021). Conserved Mechanisms, Novel Anatomies: The Developmental Basis of Fin Evolution and the Origin of Limbs. Diversity.

[81] Zakany J., Duboule D. (2007). The Role of Hox Genes during Vertebrate Limb Development. Curr. Opin. Genet. Dev..

[82] Chan Y.F., Marks M.E., Jones F.C., Villarreal G., Shapiro M.D., Brady S.D., Southwick A.M., Absher D.M., Grimwood J., Schmutz J. (2010). Adaptive Evolution of Pelvic Reduction in Sticklebacks by Recurrent Deletion of a Pitxl Enhancer. Science.

[83] Ma L., Gore A.V., Castranova D., Shi J., Ng M., Tomins K.A., van der Weele C.M., Weinstein B.M., Jeffery W.R. (2020). A Hypomorphic Cystathionine β-Synthase Gene Contributes to Cavefish Eye Loss by Disrupting Optic Vasculature. Nat. Commun..

