# Supplementary material for: Genomic Signatures of Independently Evolved Walking-Associated Locomotion in Fishes
Source: Biology (Basel). 2026 Nov 3;15(21):1990. doi: 10.3390/biology15211990 (PMC13761534; doi:10.3390/biology15211990)
Supplement: Supplementary file 1 [file biology-15-01990-s001.zip › Supplementary Figures.pdf]

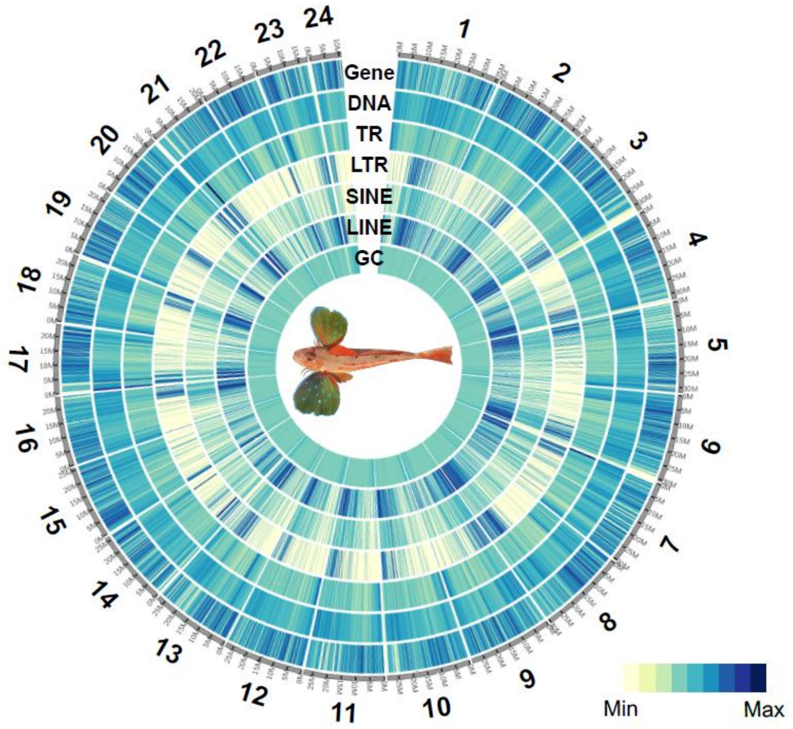

Figure S1. Genomic landscape of the spiny red gurnard (*Chelidonichthys spinosus*). Circos plot illustrating the distribution of various genomic features across the 24 chromosomes of the spiny red gurnard. The tracks from outside to inside represent chromosomes (1–24), gene density, DNA transposons, tandem repeats (TR), LTR retrotransposons, SINEs, LINEs, and GC content, respectively. The color scale for the inner tracks ranges from minimum (Min, yellow/light blue) to maximum (Max, dark blue) density values.

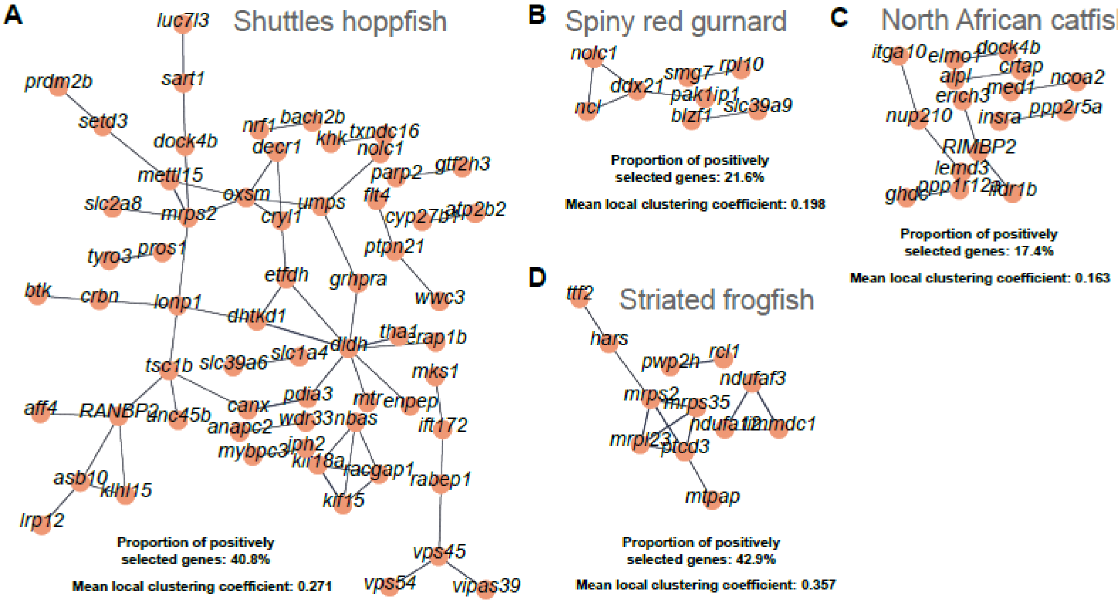

Figure S2. Species-specific protein-protein interaction (PPI) networks of positively selected genes (PSGs). PPI networks were constructed for the individual walking-associated lineages based on their respective PSGs. (A) Shuttles hoppfish; (B) Spiny red gurnard; (C) North African catfish; (D) Striated frogfish. The climbing perch is not shown because its PSG number was insufficient to construct a meaningful network. The proportion of

positively selected genes mapped to the network and the mean local clustering coefficient are indicated below each panel.

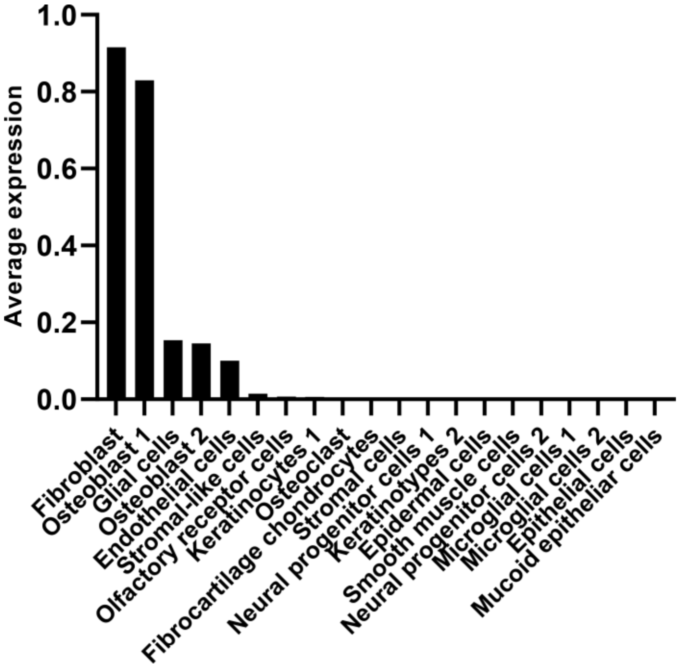

Figure S3. Single-cell expression profile of *ccdc80* across distinct cell types in the pectoral fin. Bar plot showing the average expression levels of *ccdc80* across various cell populations identified from the single-cell RNA-seq dataset of the spiny red gurnard pectoral fin. The highest expression is observed in fibroblasts and "osteoblast 1" cells.
